# Imaging α_v_β_3_ integrin expression in skeletal metastases with ^99m^Tc-maraciclatide single-photon emission computed tomography: detection and therapy response assessment

**DOI:** 10.1007/s00259-017-3926-7

**Published:** 2018-02-02

**Authors:** Gary J. R. Cook, Gurdip K. Azad, Benjamin P. Taylor, Eugene Lee, Matthew S. Morrison, Simon Hughes, Stephen Morris, Sarah Rudman, Simon Chowdhury, Vicky Goh

**Affiliations:** 1 Cancer Imaging Department, School of Biomedical Engineering and Imaging Sciences, King’s College London, London, UK; 2 Nuclear Medicine Department, Guy’s and St Thomas’ Hospitals NHS Trust, London, UK; 3 Uro-oncology Department, Guy’s and St Thomas’ Hospitals NHS Trust, London, UK; 4 GE Healthcare, Medical Diagnostics, The Grove Centre, Amersham, UK

**Keywords:** Prostate cancer, Bone metastases, ^99m^Tc-Maraciclatide, Osteoclast

## Abstract

**Purpose:**

Osteoclast activity is an important factor in the pathogenesis of skeletal metastases and is a potential therapeutic target. This study aimed to determine if selective uptake of ^99m^Tc-maraciclatide, a radiopharmaceutical targeting α_v_β_3_ integrin, occurs in prostate cancer (PCa) bone metastases and to observe the changes following systemic therapy.

**Methods:**

The study group comprised 17 men with bone-predominant metastatic PCa who underwent whole-body planar and single-photon emission computed tomography/computed tomography (SPECT/CT) imaging with ^99m^Tc-maraciclatide before (*n* = 17) and 12 weeks after (*n* = 11) starting treatment with abiraterone. Tumour to normal bone (T:N) ratios, tumour to muscle (T:M) ratios and CT Hounsfield units (HU) were measured in up to five target metastases in each subject. An oncologist blinded to study scans assessed clinical responses up to 24 weeks using conventional criteria.

**Results:**

Before treatment, metastases showed specific ^99m^Tc-maraciclatide accumulation (mean planar T:N and T:M ratios 1.43 and 3.06; SPECT T:N and T:M ratios 3.1 and 5.19, respectively). Baseline sclerotic lesions (389–740 HU) showed lower T:M ratios (4.22 vs. 7.04, *p* = 0.02) than less sclerotic/lytic lesions (46–381 HU). Patients with progressive disease (PD; *n* = 5) showed increased planar T:N and T:M ratios (0.29 and 12.1%, respectively) and SPECT T:N and T:M ratios (11.9 and 20.2%, respectively). Patients without progression showed decreased planar T:N and T:M ratios (0.27 and −8.0%, *p* = 1.0 and 0.044, respectively) and SPECT T:N and T:M ratios (−21.9, and −27.2%, *p* = 0.3 and 0.036, respectively). The percentage change in CT HU was inversely correlated with the percentage change in SPECT T:M ratios (*r* = −0.59, *p* = 0.006).

**Conclusions:**

^99m^Tc-maraciclatide accumulates in PCa bone metastases in keeping with increased α_v_β_3_ integrin expression. Greater activity in metastases with lower CT density suggests that uptake is related to osteoclast activity. Changes in planar and SPECT T:M ratios after 12 weeks of treatment differed between patients with and without PD and ^99m^Tc-maraciclatide imaging may be a potential method for assessing early response.

## Introduction

Skeletal metastases are common in patients with prostate cancer (PCa) and are associated with significant morbidity and skeletal-related events [1]. However, effective systemic treatment is available leading to improvements in overall survival of several months to years [2]. This is longer than in most other types of cancer that have metastasized to the skeleton, and the management of PCa bone metastases therefore has a significant impact on healthcare resources [3]. It is recognized that conventional imaging, including ^99m^Tc-diphosphonate bone scintigraphy or computed tomography (CT), is poor at determining treatment response or nonresponse at an early time point when patients can undergo therapeutic transition to second-line therapy that may be more effective, and can be saved from side effects of ineffective treatments [4, 5].

Whilst bone metastases from PCa are predominantly osteoblastic in nature, leading to sclerotic radiological morphology, it is known that they also demonstrate increased osteoclast activity that may be a major contributor to morbidity (e.g. pathological fracture) and which may be a therapeutic target for drugs including RANK ligand inhibitors (e.g. denosumab) and osteoclast inhibitors (e.g. bisphosphonates), that can decrease skeletal-related events [2]. Osteoclast activity is therefore a relevant process in the pathophysiology of bone metastases and may provide a useful target for detection and response assessment by imaging probes. Osteoclasts express higher levels of the α_v_β_3_ integrin than any other cell types in the body [6] and this integrin heterodimer is involved in the adhesion of osteoclasts to the bone matrix [7]. It strongly binds the Arg-Gly-Asp (RGD) motif, which when radiolabelled, has been used to successfully image α_v_β_3_ integrin expression related to osteoclast activity in animal models [8, 9]. ^99m^Tc-Maraciclatide (^99m^Tc-NC100692) is a cyclic peptide with a RGD binding site with high affinity for the α_v_β_3_ integrin in vitro and in vivo, and has been investigated for detection of α_v_β_3_ integrin overexpression in angiogenesis [10, 11].

In this study our hypotheses were that ^99m^Tc-maraciclatide specifically accumulates in PCa bone metastases and that changes in uptake correlate with treatment response to systemic therapy. Our aims were to measure uptake of ^99m^Tc-maraciclatide in patients with de-novo or progressive bone metastases from PCa, to correlate uptake and changes in uptake with CT density (Hounsfield units, HU), as a surrogate for the underlying osteoblastic and osteoclastic activity, and to determine if changes in uptake at 12 weeks could act as a marker of treatment response.

## Materials and methods

The study group comprised 17 consecutive men (mean age 73.4 years) with de-novo or progressive bone-predominant metastatic PCa scheduled for treatment with abiraterone. Informed consent was obtained from all individual participants included in the study. Ethical and radiation committee (ARSAC) approvals were obtained for the study. Six patients did not undergo 12-week imaging, in two due to patient choice and in four due to lack of tracer availability. In 11 patients who had follow-up ^99m^Tc-maraciclatide imaging at 12 weeks, a clinical oncologist blinded to the ^99m^Tc-maraciclatide imaging defined the clinical response to abiraterone using conventional clinical (including pain score), biochemical (prostate-specific antigen, alkaline phosphatase) and imaging results (CT and bone scan) up to 24 weeks after the start of therapy. Clinical response was recorded as progressive disease (PD) or non-PD, i.e. stable disease, partial response or complete response.

Patients underwent whole-body planar scintigraphy 45 min after injection of 740 MBq ^99m^Tc-maraciclatide using a dual-headed gamma camera (Philips, Cleveland, OH) with low-energy high-resolution collimators and a 20% energy window centred on 140 keV, before and 12 weeks after starting abiraterone. An additional single-photon emission CT/CT (SPECT/CT) scan of a target lesion was then acquired. The acquisition parameters for the SPECT component were 20 s/frame and 128 projections over 360° and the images were reconstructed with an ordered subsets expectation maximization algorithm with five iterations over eight subsets. The acquisition parameters for the CT component were 100 mAs/slice, 120 kV, pitch 1.188 and rotation time 0.75 s.

Regions of interest (ROI) were drawn manually around up to five target lesions per patient on anterior and posterior planar scans to calculate the geometric mean activity. If a lesion could only be visualized on either the anterior or the posterior view, then only this ROI was used for subsequent analysis. A contralateral ROI of the same area was placed over a normal area of bone and if the lesion was in the midline, e.g. the spine, then the nearest normal midline vertebra was used to define an area of normal skeletal uptake. A tumour to normal bone (T:N) ratio was calculated for each metastasis. ROIs were also placed over muscle in the right lateral thigh to calculate a geometric mean muscle activity and hence a tumour to muscle (T:M) ratio. For SPECT imaging T:N and T:M ratios were calculated for each of the five lesions within the field of view with counts averaged for all the slices in which tumour activity was visible. For muscle activity the erector spinae muscle was used when the lateral thigh was not in the field of view. The same lesions were analysed in the 11 patients who had 12-week scans and the mean percentage change in each ^99m^Tc-maraciclatide uptake ratio was also recorded. Mean CT HU were recorded for each lesion on the corresponding CT component of the SPECT/CT acquisition on pretreatment and 12-week scans. No attempt was made to measure an index of global skeletal involvement as previously reported for bone scintigraphy [12], as planar T:N ratios and normal skeletal uptake are lower than those from standard bone scintigraphy, precluding this approach.

Changes in uptake ratios were compared between patients with clinical PD and those with non-PD and the significance of differences in uptake ratios and in percentage change in uptake ratios between those below and above the median baseline CT HU value and between those below and above the median percentage change in CT HU, respectively, were evaluated using Student’s *t* test or the Mann-Whitney *U* test depending on whether or not the data were normally distributed as tested by the Shapiro-Wilk test. Spearman’s test was used to determine correlations between percentage change in CT HU and percentage change in ^99m^Tc-maraciclatide uptake. Statistical tests were performed with IBM SPSS Statistics, version 24.

## Results

**Fig. 1:**
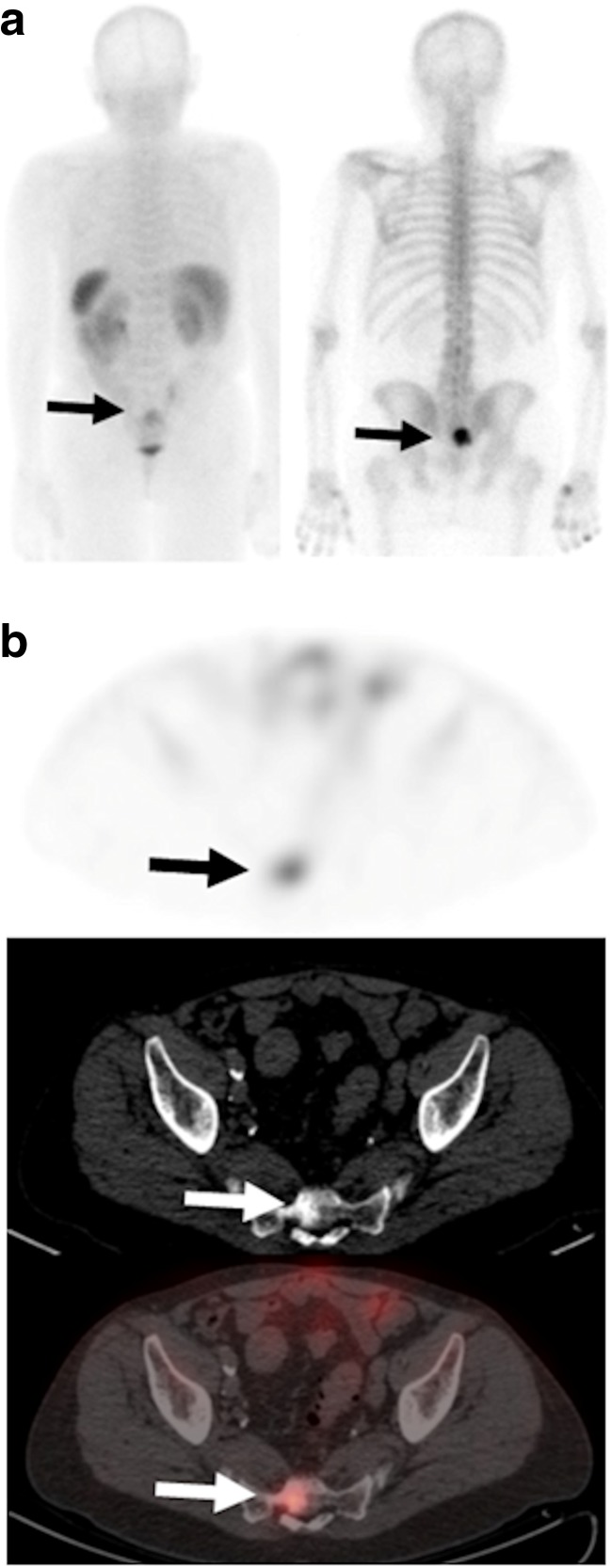
**a** ^99m^Tc-maraciclatide posterior planar image (*left*) shows uptake in a sacral metastasis (*arrows*; T:N ratio 1.81, T:M ratio 4.79) that is also demonstrated on the ^99m^Tc-methylene diphosphonate bone scan (*right*). **b** ^99m^Tc-maraciclatide uptake is visible on the axial SPECT image (*top*) and the fused SPECT/CT image (*bottom*; T:N ratio 8.0, T:M ratio 10.0), and is visible on the CT bone windows (*centre*; HU = 381)

**Fig. 2:**
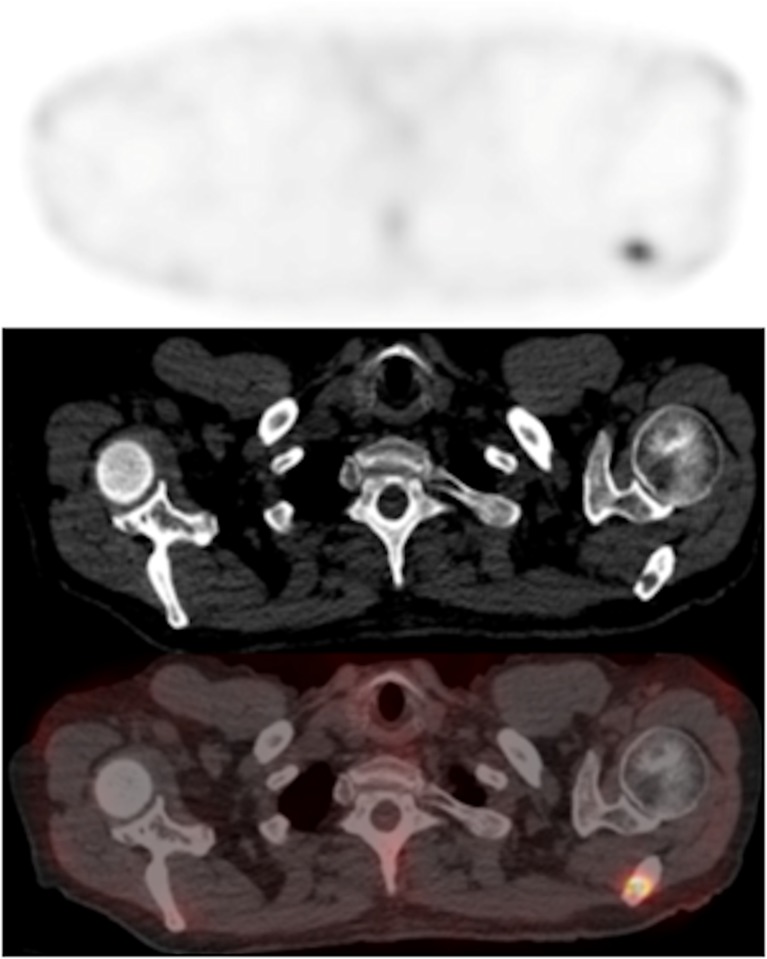
Axial SPECT image (*top*) and axial fused SPECT/CT image (*bottom*) show ^99m^Tc-maraciclatide uptake in a left scapula metastasis (T:N ratio 8.25, T:M ratio 5.91) that appears lytic on the CT image (*centre*; HU = 46)

In the 11 patients who underwent baseline and 12-week imaging, the clinical reference standard indicated PD in five patients and non-PD in six patients (stable disease in five, treatment response in one). There was no significant difference in percentage change in serum calcium or alkaline phosphatase levels between those with PD (2.4% and 23.6%, respectively) and those with non-PD (−0.7% and 10.4%, respectively). Metastatic bone lesions showed accumulation of ^99m^Tc-maraciclatide with mean planar T:N and T:M ratios of 1.43 (± 0.27) and 3.06 (± 0.83), respectively, and SPECT T:N and T:M ratios of 3.1 (± 1.7) and 5.19 (± 2.77), respectively, before treatment in the 17 patients (Fig. 1). In these scans, the most sclerotic lesions (389–740 HU, mean 583 HU) showed lower ^99m^Tc-maraciclatide T:M ratios (4.22 vs. 7.04, *p* = 0.02) than the less sclerotic or lytic lesions (46–381 HU, mean 223 HU; Fig. 2).

**Fig. 3:**
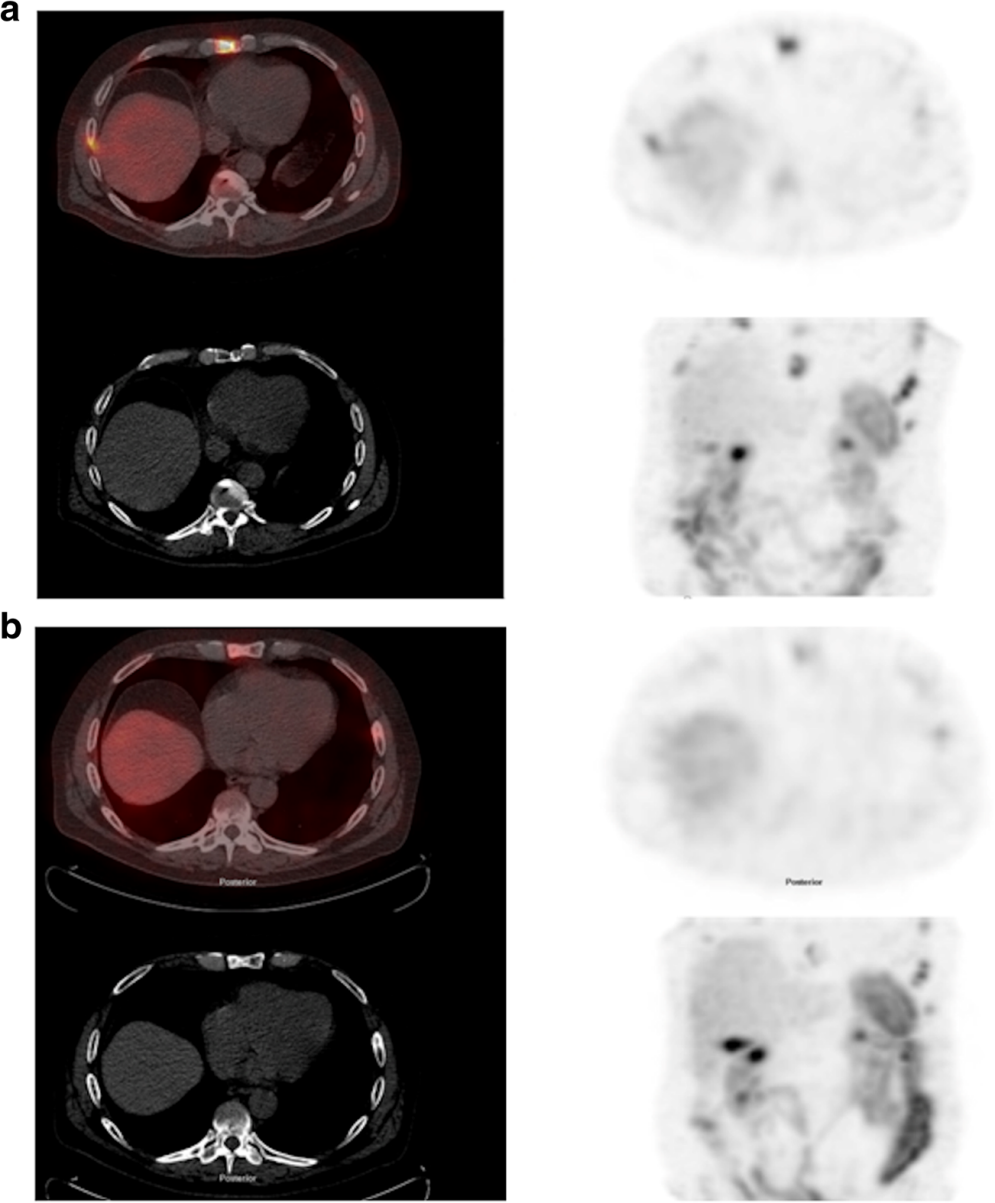
Fused axial SPECT/CT image (*top left*), CT image (*bottom left*), axial SPECT image (*top right*) and coronal maximum intensity image (*bottom left*) show a sternal lesion in a patient with a treatment response: **a** before treatment (T:N ratio 8.66, T:M ratio 11.97, HU = 155), **b** 12 weeks after starting treatment (T:N ratio 4.42, T:M ratio 5.18, HU = 413)

Five patients with PD showed a mean percentage increase in planar T:N and T:M ratios and SPECT T:N and T:M ratios (0.29, 12.1, 11.9 and 20.2%, respectively) whereas six patients with non-PD showed no change or a reduction in T:M ratios (0.27, −8.0, −21.9, −27.2%, respectively; Fig. 3). Changes in planar and SPECT T:M ratios were significantly different between patients with PD and those with non-PD (*p* = 0.044 and 0.036, respectively) whereas changes in T:N ratios were not significantly different (*p* = 1.0 and 0.3, respectively). Percentage change in CT HU in individual lesions between baseline and 12 weeks showed an inverse correlation with percentage change in SPECT T:M ratios (*r* = −0.59, *p* = 0.006). Percentage change in SPECT T:M ratios in lesions with an increase in CT HU (5.8% to 290.9%) were significantly different from those with a decrease in CT HU (−7.9 to −67.1%) with median percentage changes of −30.9% and 3.9%, respectively (*p* = 0.016).

## Discussion

This study showed that ^99m^Tc-maraciclatide, a tracer that is known to target the α_v_β_3_ integrin [10], specifically accumulates in metastatic bone lesions in patients with PCa. The uptake of ^99m^Tc-maraciclatide (SPECT T:M ratio) was higher in the untreated lowest density bone metastases than in those with higher density, as measured by CT HU, suggesting that uptake is related to the degree of osteoclast activity. Previous in vitro experiments with an alternative positron emission tomography (PET) α_v_β_3_ integrin targeting tracer, ^64^Cu-CB-TE2A-c(RGDyK), have shown specific uptake in osteoclasts and a correlation with osteoclast numbers in a mouse model of parathyroid hormone-induced osteolysis in the calvaria [8]. Similarly, the same PET tracer has shown specific uptake in a mouse model of osteolytic bone metastases [9], and a different PET tracer, ^68^Ga-DOTA-E-[c(RGDfK)]_2_, has shown specific accumulation in a rat model of breast cancer bone metastasis [13]. In humans, a different ^99m^Tc-RGD tracer (^99m^Tc-3PRGD2) has been shown to detect bone metastases from lung cancer with high sensitivity and with better specificity than ^99m^Tc-methylene diphosphonate scintigraphy [14]. A PET tracer, ^18^F-galacto-RGD, has been found to accumulate in metastatic PCa with a T:M ratio of 2.8 ± 1.3 leading to the detection of 58 out of 74 metastases in 12 patients [15].

In our study, patients who had clinical PD by 24 weeks showed significantly different percentage changes in planar and SPECT T:M ratios at 12 weeks (12.1% and 20.2% increases, respectively) compared with those with non-PD (−8.0% and −27.2% decreases, *p* = 0.044 and 0.036, respectively) suggesting that ^99m^Tc-maraciclatide may be useful for determining early treatment response. A difference in percentage change in ^99m^Tc-maraciclatide uptake (SPECT T:M ratio) was also seen between lesions that showed an increase in CT HU and those that showed a decrease (*p* = 0.016), and the inverse correlation also suggests that ^99m^Tc-maraciclatide activity may be related to underlying changes in the bone microenvironment following treatment, including changes in osteoclast activity. These data are supported by the previously mentioned preclinical study of osteolytic metastases in a transgenic mouse model that showed reductions in uptake of ^64^Cu-CB-TE2A-c(RGDyK) following treatment with an osteoclast inhibitor, the bisphosphonate zolendronate [9]. Human skeletal diseases that are known to exhibit greater osteoclast activity than PCa, such as multiple myeloma [16] or periprosthetic osteolysis [17], would provide relevant clinical models to further test this hypothesis.

The main limitation of this study is that we had no direct measurement of the proportion of uptake of ^99m^Tc-maraciclatide that was specific to osteoclast activity or what degree of uptake may have been tumour-specific or endothelial/angiogenesis-specific. Indeed it is known that PCa cell lines derived from bone metastases express the α_v_β_3_ integrin [18] and we cannot exclude an effect on baseline measurements from previous therapy in the subset of our patients recruited with PD following previous treatment. Nevertheless, the inverse correlations we observed with CT density measured in HUs that is known to be related to osteoclastic and osteoblastic phenotypes before treatment and, in particular, the changes with treatment, support the hypothesis that ^99m^Tc-maraciclatide uptake is related to osteoclast activity through targeting the α_v_β_3_ integrin. Planar T:N and T:M ratios are prone to greater measurement error than SPECT ratios but the planar ratios are nevertheless of interest in this first study of ^99m^Tc-maraciclatide imaging of skeletal metastases in PCa in providing a quantitative description of uptake and a comparator for future studies. Secondly, abiraterone is not known to have a direct anti-osteoclastic effect and it is likely that the changes in ^99m^Tc-maraciclatide uptake we observed in bone metastases were in part the result of a secondary downstream reduction in osteoclast activity following the antitumour action of abiraterone caused by inhibition of androgen synthesis [19].

### Conclusions

^99m^Tc-maraciclatide accumulates in bone metastases from PCa in keeping with increased expression of α_v_β_3_ integrin, and shows an inverse correlation with CT density, suggesting a relationship with osteoclast activity. In addition, changes in ^99m^Tc-maraciclatide uptake after 12 weeks of systemic therapy with abiraterone differ between patients with subsequent PD and non-PD and this may therefore be a potential method for predicting clinical response that deserves further study.
